# Adenoviral Delivery of Angiotensin-(1-7) or Angiotensin-(1-9) Inhibits Cardiomyocyte Hypertrophy via the Mas or Angiotensin Type 2 Receptor

**DOI:** 10.1371/journal.pone.0045564

**Published:** 2012-09-20

**Authors:** Monica Flores-Muñoz, Bruno M. D. C. Godinho, Abdulaziz Almalik, Stuart A. Nicklin

**Affiliations:** Institute of Cardiovascular and Medical Sciences, University of Glasgow, Glasgow, United Kingdom; French National Centre for Scientific Research, France

## Abstract

The counter-regulatory axis of the renin angiotensin system peptide angiotensin-(1-7) [Ang-(1-7)] has been identified as a potential therapeutic target in cardiac remodelling, acting via the mas receptor. Furthermore, we recently reported that an alternative peptide, Ang-(1-9) also counteracts cardiac remodelling via the angiotensin type 2 receptor (AT_2_R). Here, we have engineered adenoviral vectors expressing fusion proteins which release Ang-(1-7) [RAdAng-(1-7)] or Ang-(1-9) [RAdAng-(1-9)] and compared their effects on cardiomyocyte hypertrophy in rat H9c2 cardiomyocytes or primary adult rabbit cardiomyocytes, stimulated with angiotensin II, isoproterenol or arg-vasopressin. RAdAng-(1-7) and RAdAng-(1-9) efficiently transduced cardiomyocytes, expressed fusion proteins and secreted peptides, as demonstrated by western immunoblotting and conditioned media assays. Furthermore, secreted Ang-(1-7) and Ang-(1-9) inhibited cardiomyocyte hypertrophy (Control = 168.7±8.4 µm; AngII = 232.1±10.7 µm; AngII+RAdAng-(1-7) = 186±9.1 µm, RAdAng-(1-9) = 180.5±9 µm; P<0.05) and these effects were selectively reversed by inhibitors of their cognate receptors, the mas antagonist A779 for RAdAng-(1-7) and the AT_2_R antagonist PD123,319 for RAdAng-(1-9). Thus gene transfer of Ang-(1-7) and Ang-(1-9) produces receptor-specific effects equivalent to those observed with addition of exogenous peptides. These data highlight that Ang-(1-7) and Ang-(1-9) can be expressed via gene transfer and inhibit cardiomyocyte hypertrophy via their respective receptors. This supports applications for this approach for sustained peptide delivery to study molecular effects and potential gene therapeutic actions.

## Introduction

The renin-angiotensin system (RAS) is recognized for its systemic actions, however the presence of RAS components in specific tissues (e.g. heart, brain, kidney), suggests the presence of a local RAS. Furthermore, a counter-regulatory axis of the RAS exists, which functions mainly via angiotensin converting enzyme 2 (ACE2)/angiotensin (Ang)-(1-7)/mas and inhibits many detrimental cardiovascular disease phenotypes [1], [2]. Ang-(1-7) has been shown to antagonise pathological actions such as cardiac hypertrophy and fibrosis through the receptor mas [1], [3]. Most recently we reported that Ang-(1-9), a poorly characterised peptide not previously reported as a receptor agonist, also had anti-hypertrophic effects on angiotensin II (AngII)-induced cardiomyocyte hypertrophy, as a functional ligand at the angiotensin type 2 receptor (AT_2_R) [4]. Moreover, we also demonstrated that Ang-(1-9) reduced cardiac fibrosis in stroke prone spontaneously hypertensive rats through the AT_2_R [5]. These studies highlight the potential for therapeutic application of Ang-(1-7) and Ang-(1-9) in cardiovascular disease applications. Though active angiotensin peptides are generated extracellularly in the plasma via renin mediated cleavage of angiotensinogen to angiotensin I, followed by ACE-mediated cleavage to AngII, methods which enable their expression through gene transfer approaches are available. Transgenic expression of AngII and Ang-(1-7) can be mediated through the use of synthetic fusion protein expression cassettes which are expressed intracellularly and result in cleavage and secretion of active peptides. Such approaches have been utilised to demonstrate organ-specific effects of individual angiotensin peptides in the heart, kidney and brain [6]–[14]. Gene therapy approaches have also been reported for Ang-(1-7) in models of both myocardial infarction and diabetic retinopathy using viral vector-mediated gene transfer, highlighting their potential in this setting [15], [16]. Here, we have generated adenoviral (Ad) vectors encoding fusion proteins expressing Ang-(1-7) or Ang-(1-9) and compared their effects in models of cardiomyocyte hypertrophy. We report that adenoviral gene transfer can be used to express different angiotensin peptides and it can be shown that these peptides are secreted from cells and maintain the receptor-specific interactions that have been reported for the endogenous peptides. This highlights the general applicability of this approach and importantly for the first time demonstrates that Ang-(1-9) can be expressed via adenoviral gene transfer and mediate functional effects at the AT_2_R.

## Results

### Generation of RAdAng-(1-7) and RAdAng-(1-9)

**Figure 1:**
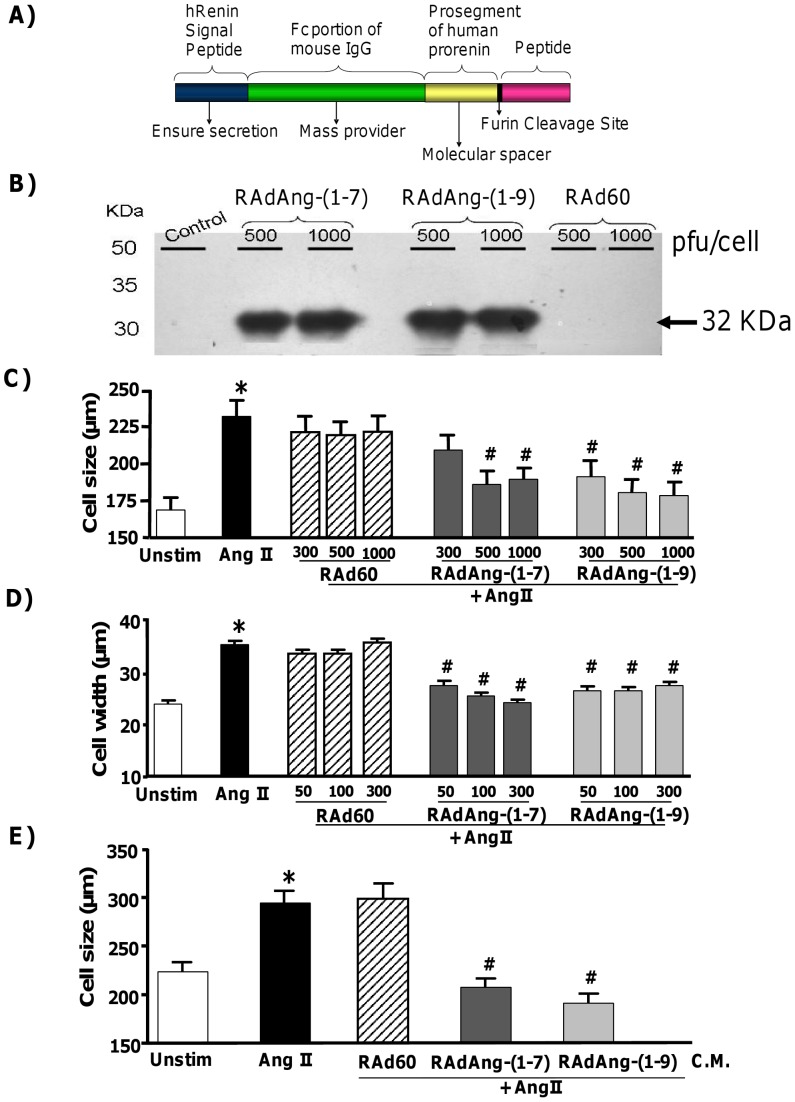
Detection of fusion protein expression and functional assessment of RAdAng-(1-7) and RAdAng-(1-9). (A) Schematic of fusion protein, consisting of a renin signal peptide to ensure secretion, murine IgG to provide mass for efficient production of the protein, a furin protease cleavage domain (to invoke peptide release), and each peptide [6]. (B) H9c2 cardiomyocytes were transduced with 500 or 1000 pfu/cell of RAdAng-(1-7), or RAdAng-(1-9), or RAd60 lysed after 48 h and subjected to electrophoresis. Fusion protein expression was detected by western immunoblotting using a α-IgG2b antibody. kDa = kilodaltons. (C) H9c2 cardiomyocytes were transduced with RAdAng-(1-7), RAdAng-(1-9) or RAd60 at 500 and 1000 pfu/cell 24 h before AngII addition. Following 96 h incubation cells were fixed, stained with crystal violet and cell size measured. *p<0.01 vs. unstimulated cells; ^#^p<0.05 vs. AngII stimulated cells. (D) Freshly isolated left ventricular adult rabbit primary cardiomyocytes were transduced with RAdAng-(1-7), RAdAng-(1-9) or RAd60 (50, 100 and 300 pfu/cell) 1 h before AngII (500 nM) addition. After 24 h cell width was measured. *p<0.01 vs. unstimulated cells; ^#^p<0.01 vs. AngII stimulation. (E) HeLa cells were transduced with RAdAng-(1-7), RAdAng-(1-9) or RAd60 (100 pfu/cell) and incubated for 48 hours. Culture media from HeLa transduced cells (C.M; conditioned media) was transferred to H9c2 cardiomyocytes and incubated for 30 minutes before AngII (100 nM) addition. 96 hours later cells were fixed, stained with crystal violet and cell size measured. *p<0.01 vs. unstimulated cells; ^#^p<0.01 vs. AngII stimulated cells.

The fusion protein expression cassette consists of a signal peptide, an IgG molecule linked to Ang-(1-7) or Ang-(1-9) and a cleavage site for furin protease enabling active peptides to be secreted (Figure 1A). Western immunoblotting of Ad transduced H9c2 cardiomyocytes demonstrated expression of each fusion protein with a size of 32 kDa as expected (Figure 1B).

### Assessment of the Effects of RAdAng-(1-7) and RAdAng-(1-9) Delivery on Cardiomyocyte Hypertrophy

First we assessed the efficiency of Ad transduction into H9c2 and rabbit primary cardiomyocytes using a reporter gene (β-galactosidase) expressing Ad vector. Doses of 500 and 1000 pfu/cell in H9c2 cells and 50 and 100 pfu/cell in rabbit cardiomoytes produced approximately 50 and a 100% transduction, respectively (data not shown). To assess the effects of RAdAng-(1-7) and RAdAng-(1-9) on cardiomyocyte hypertrophy we used *in vitro* stimulation with AngII as described previously [4], [17], [18]. AngII induced cardiomyocyte hypertrophy as expected (Figure 1C). Adenoviral gene delivery *per se* did not affect cell size as RAd60 transduced cells were not significantly different to AngII stimulated cells. However, at 500 and 1000 pfu/cell both RAdAng-(1-7) and RAdAng-(1-9) inhibited AngII-induced hypertrophy (AngII = 232.1±10.7 µm; RAdAng-(1-7) 300 pfu = 209.2±10.4 µm; RAdAng-(1-7) 500 pfu = 186±9.1 µm; RAdAng-(1-7) 1000 pfu = 189.5±7.5 µm; RAdAng-(1-9) 300 pfu = 191.4±10.7 µm; RAdAng-(1-9) 500 pfu = 180.5±9.0 µm; RAdAng-(1-9) 1000 pfu = 178.5±9.1 µm; p<0.05), indicating that each RAd was functional and antagonized AngII-induced hypertrophy. Next, RAdAng-(1-7) and RAdAng-(1-9) were assessed in adult rabbit left ventricular primary cardiomyocytes. Similarly to what was observed previously with exogenous peptides [4], RAdAng-(1-7) and RAdAng-(1-9) were able to inhibit AngII-induced hypertrophy by preventing the AngII-stimulated increase in cell width which is indicative of concentric hypertrophic growth (Figure 1D).

### Assessment of Peptide Secretion and Function from RAdAng-(1-7) and RAdAng-(1-9) Transduced Cells

**Figure 2:**
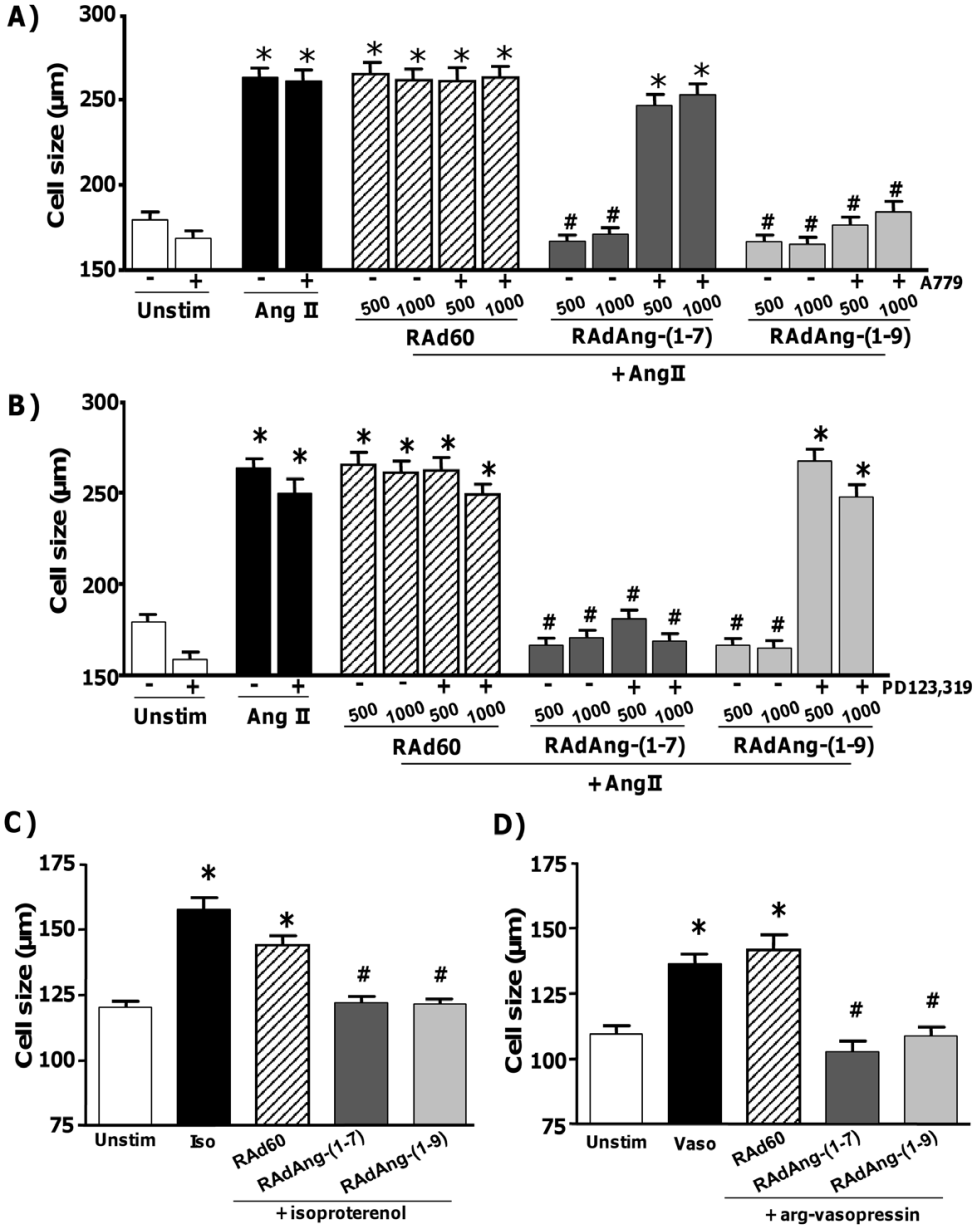
Effect of Mas and AT_2_R antagonism on AngII-stimulated H9c2 cardiomyocyte hypertrophy following RAdAng-(1-7), or RAdAng-(1-9) transduction. H9c2 cardiomyocytes were transduced with RAdAng-(1-7), RAdAng-(1-9) or RAd60 (negative control) +/− (A) the Mas antagonist A779 (10 µM) or (B) the AT_2_R antagonist PD123,319 (500 nM) 24 h before AngII addition. Cells were incubated for 96 h before fixing, staining and measurement of cell size. *p<0.01 vs. unstimulated cells, ^#^p<0.001 vs. AngII stimulated cells. H9c2 cardiomyocytes were transduced with RAdAng-(1-7), RAdAng-(1-9) or RAd60 at 500 pfu/cell 24 h before addition of (C) 1 µM isoproterenol (Iso) or (D) 1 µM arg-vasopressin (vaso). Cell size was measured at 96 h. *p<0.05 vs. unstimulated cells, ^#^p<0.05 vs. isoproterenol or arg-vasopressin stimulated cells.

To demonstrate that Ang-(1-7) and Ang-(1-9) were secreted from the cells in an active form a conditioned media assay was utilised. HeLa cells were transduced with 100 pfu/cell of either RAdAng-(1-7), RAdAng-(1-9) or RAd60 and incubated in serum free media for 48 hours. Conditioned media was then transferred to AngII-stimulated H9c2 cardiomyocytes (Figure 1E). Transfer of conditioned media from RAd60 transduced HeLa cells to AngII-stimulated H9c2 cardiomyocytes had no effect on hypertrophy. However, transfer of conditioned media from RAdAng-(1-7) or RAdAng-(1-9) transduced HeLa cells to AngII-stimulated cardiomyocytes inhibited hypertrophy (p<0.01), thus confirming each RAd expressed the fusion protein and secreted the active peptide and produced effects equivalent to those observed previously with direct peptide incubation [4]. To demonstrate that the peptides expressed and secreted via gene transfer functioned similarly to exogenous peptides, antagonists of mas and AT_2_R function were assessed. In similarity to observations with exogenous peptides [3], [4], addition of A779 abolished the anti-hypertrophic effect of RAdAng-(1-7), while RAdAng-(1-9) was able to inhibit AngII-induced hypertrophy in the presence of A779 (Figure 2A). Addition of PD123,319 did not block the antihypertrophic effect of Ang-(1-7), but, completely eliminated those of RAdAng-(1-9) (Figure 2B).

### Assessment of RAdAng-(1-7) and RAdAng-(1-9) in Cardiomyocyte Hypertrophy Induced with Different Stimuli

To provide evidence that the antihypertrophic effects of RAdAng-(1-7) or RAdAng-(1-9) delivery were not confined to AngII stimulation, effects in cardiomyocyte hypertrophy induced by isoproterenol and arg-vasopressin were measured (Figure 2C–D). Both RAdAng-(1-7) and RAdAng-(1-9) were able to inhibit isoproterenol and arg-vasopressin induced hypertrophy. This verified that gene delivery of Ang-(1-7), or Ang-(1-9) was able to inhibit cardiomyocyte hypertrophy induced with different stimuli relevant to cardiovascular disease.

## Discussion

We have generated adenoviral vectors which selectively over-express Ang-(1-7) or Ang-(1-9). RAdAng-(1-7) and RAdAng-(1-9) and transduce neonatal rat cardiomyoycte cell lines and adult primary rabbit cardiomyocytes and secrete RAS peptides. Transducing each respective cell type with viral vector doses which achieved 50 or 100% transduction efficiency resulted in secreted peptides inhibiting cardiomyocyte hypertrophy in the same manner as described for exogenous peptides, via mas and AT_2_R respectively [3], [4], confirming over-expression and release of active peptides via adenoviral transduction.

Currently, there is great interest in studying the counter-regulatory ACE2/Ang-(1-7)/mas axis and our own recent publications have also highlighted the importance of studying other peptides such as Ang-(1-9), which may have, as yet, relatively unexplored functions [4], [5]. Most studies of angiotensin peptide function utilise systemic delivery of peptides via osmotic minipumps, however, transgenic models directing tissue-specific production of angiotensin peptides to avoid confounding systemic effects have also been developed [6]–[14]. These studies have proved invaluable in our understanding of endogenous tissue-specific RAS. However, the possibility of developmental abnormalities caused by embryonic over-expression following transgenic model generation cannot be discounted. Such effects may explain the discrepancies between transgenic studies and gene over-expression studies. For example, ACE2 null mice have been reported to have increased blood pressure with no cardiac changes [2] while ACE2 overexpression by adeno-associated virus (AAV) 6-mediated gene delivery leads to severe cardiac fibrosis [19]. Furthermore, Ang-(1-7) has previously been investigated as a gene therapeutic approach utilising viral vector mediated delivery. In a rat model of myocardial ischaemia lentiviral gene transfer of Ang-(1-7) 5 weeks before induction of myocardial infarction prevented decreased myocardial performance as indicated by improvements in fractional shortening and decreased myocardial wall thinning [15]. In a model of diabetic retinopathy local ocular delivery of adeno-associated virus (AAV) expressing Ang-(1-7) prevented diabetes-induced retinal vascular damage and inflammation [16]. Furthermore, adenoviral gene transfer of AngII to vascular smooth muscle cells *in vitro* resulted in secretion of AngII and functional effects on smooth muscle cell hypertrophy [20]. These studies highlight the potential for gene transfer of angiotensin peptides for molecular investigations and therapeutic approaches and our study builds on these findings.

Adenoviral vectors mediate acute overexpression of genes in adult cardiomyocytes *in vitro* and *in vivo* which may be applicable for gene therapy applications in acute conditions such as myocardial infarction (MI) and furthermore, their tropism is efficient for other cell types in other organs such as kidney, brain and skeletal muscle, which are also important to study tissue-specific RAS effects. Moreover, for long term expression of peptides in the heart AAV, particularly serotypes such as 1, 6 and 9 [21], can produce stable expression in myocardium following non-invasive intravenous delivery which may be beneficial to modulate long term detrimental remodelling in the heart following an MI, in order to counter progression to heart failure. Therefore, there are a range of delivery vectors which will enable assessment of Ang-(1-7) and Ang-(1-9) in gene therapy approaches.

In summary, our data demonstrates that Ang-(1-7) and Ang-(1-9) can be expressed via adenoviral gene transfer and inhibit cardiomyocyte hypertrophy via their respective receptors. Future work with these vectors is required *in vivo* to assess potential therapeutic effects induced following cardiac-selective delivery.

## Materials and Methods

### Production of Recombinant Adenoviruses (RAd)

RAds expressing Ang-(1-7) [RAdAng-(1-7)] or Ang-(1-9) [RAdAng-(1-9)] were generated by modifying pBluescript-pre-fc-proAngII [22] (Figure 1A). The furin cleavage site and AngII encoding nucleotides were excised via *Bg*lII and *Eco*RI restriction and the vector backbone gel purified. Oligonucleotides encoding a furin cleavage site, Ang-(1-7) or Ang-(1-9) flanked by *Bg*lII and *Eco*RI restriction sites were commercially synthesised: Ang-(1-7) Forward 5′-GATCTCGCGTACGCACTAAACGCGACCGGGTGTACATACACCCCTGAG-3′, Reverse 3′-AGCGCATGCGTGATTTGCGCTGGCCCACATGTATGTGGGGACTCTTAA-5′; Ang-(1-9) Forward 5′-GATCTCGCGTACGCACTAAACGCGACCGGGTGTACATACACCCCTTCC ACTGAG-3′, Reverse 3′-AGCGCATGCGTGATTTGCGCTGGCCCACATGTATGTGGGGA AGGTGACTCTTAA-5′. Annealed oligonucleotides encoding Ang-(1-7) or Ang-(1-9) were ligated into pBluescript-pre-fc-pro and transformed into competent *E.coli* JM109 (Promega, Southampton, UK). Sequencing confirmed their correct insertion. Next, Ang-(1-7) or Ang-(1-9) fusion protein encoding expression cassettes were excised from pBluescript-pre-fc-pro, purified and cloned into pVQ-CMV-KNpA and recombined with pacAd5 9.2–100 in 293 cells [23] to generate RAdAng-(1-7) or RAdAng-(1-9). RAd60 is a recombinant Ad vector without transgene and has been described previously [24]. Adenoviral vectors were amplified, purified and titered by standard protocols [25].

### Cardiomyocyte Hypertrophy and Adenoviral Transduction

H9c2 cardiomyocytes were obtained from ECACC (Wiltshire, UK). The isolation of primary rabbit cardiomyocytes was approved by the University of Glasgow Animal Procedures and Ethics Committee and performed in strict accordance with UK Home Office guidelines under the Animals Scientific Procedures Act 1986. Isolation was performed as previously described [4]. Expression of the fusion proteins in H9c2 cardiomyocytes transduced with 500 or 1000 plaque forming units (pfu)/cell of each RAd for 48 h was confirmed by western immunoblotting as described previously [22] using a mouse anti-IgG2b antibody (rabbit polyclonal 1:250; Abcam, Cambridge, UK), which detects expression of the IgG portion of the fusion protein (Figure 1A). Cardiomyocytes were stimulated with AngII, arginine-vasopressin or isoproterenol to induce hypertrophy as described previously [4], [17], [18]. Cell size was measured using Image ProPlus 4.1 image software (Media Cybernetics, Basingstoke, UK ). For each condition we measured 100 cells (10 fields of view in each condition). In H9c2 cardiomyocytes cell length was measured. In primary cardiomyocytes midpoint width was measured as previously described [4]. To assess RAdAng-(1-7) or RAd-(Ang-1-9) in cardiomyocyte hypertrophy H9c2 cardiomyocytes were transduced with 500 or 1000 plaque forming units (pfu)/cell and primary rabbit left ventricular cardiomyocytes with 50, 100 or 300 pfu/cell of RAdAng-(1-7), RAdAng-(1-9) or RAd60 (negative control). After 24 h incubation, cells were stimulated with AngII (100 nM; Sigma, Dorset, UK), isoproterenol (1 µM; Sigma, Dorset, UK) or arg-vasopressin (1 µM; Sigma, Dorset, UK) and incubated for 96 hours (H9c2) or 24 h (rabbit). To assess receptor use experiments were performed in the presence or absence of the Mas antagonist A779 (10 µM; Bachem, Rhein, Germany) or the AT_2_R antagonist PD123,319 (500 nM; Sigma, Dorset, UK).

### Conditioned Media Assay

To provide evidence that the peptides were secreted using a conditioned media assay. HeLa cells were transduced with 100 pfu/cell of either RAdAng1-7, RAdAng1-9 or RAd60 and incubated in serum free media for 48 hours at 37°C to allow the secreted peptides to accumulate in the media (conditioned media). The conditioned media then was collected and added to previously quiesced H9c2 cardiomyocytes 30 minutes before addition of AngII (100 nM). H9c2 cardiomyocytes were incubated for 96 hours before measuring cell size as already mentioned.

### Statistical Analysis

Experiments were performed in triplicate on 3 different occasions. Mean ± standard error of the mean (S.E.M) is presented. One way ANOVA with Bonferroni’s correction for multiple comparisons were applied and statistical difference was considered with *p* values <0.05.
